# Transactions of the Medical Society of the County of Kings

**Published:** 1865-09

**Authors:** 


BUFFALO

### Medical and Surgical Journal.

VOL. V. SEPTEMBER, 1865. No. 2.

ART. I—*Transactions of the Medical Society of the County of Kings.*

### REGULAR MEETINGS, MARCH AND JUNE, 1865.

*Action of Medicines on the Blood-Vessels.* By R. Cresson Stiles, M. D.

[Continued from August Number.]

The necessary result of the existence in the blood of a substance acting simply to relax the muscular coat of the blood vessels would be, in the first place, an increase of pressure in the arterioles and capillaries, in accordance with a well-known law of physics. This has been shown by Bernard to be the fact in several local circulations by cardiometric experiments. He has shown also that wourara, injected into the arteries of the sub-maxillary gland, produces paralysis of the motor nerves of the blood vessels, with consequent increased rapidity of the circulation and escape of the blood from the vein in jets, corresponding with the pulses of the heart.* Should these phenomena of paralysis be systemic, instead of local, general venous distension would ensue, did not the heart beat more actively and the lungs transmit the crowding blood more rapidly. But the chief stimulus to the rythmical action of the heart is its supply of blood. Even after the medulla oblongata has been severed, we can control the action of the heart by withdrawing blood from the system, and injecting it again into the the blood vessels; can thus arrest its motion, and restore it to action; or the same result can be effected bv remitting or accelerating the movements of artificial respiration, thus diminishing or increasing the flow of blood to the heart through the lungs. The

* See Journal d'Anat. et de Physiol. 1864, p. 511.

heart is the servant of the tissues and organs; it is from the capillary circulation mainly that its impulses are derived; hence, increased capillary circulation necessitates, when the links of the vital chain are still unbroken, increased action of the heart, and the flame of vital combustion may thus be fanned to fever heat.

That the common properties of muscular tissue are affected in our most characteristic febrile disorders is seen in the excessive prostration, muscular pains and weakness; in the slipping down in bed, and in the muttering delirium (one whose energies find no muscular expression;) in the difficulty of swallowing, while the clearness of the intellect shows cerebral activity not seriously disturbed; in the tympanitic condition of the bowels, and the oppressed respiration. The increased pressure in the capillaries is marked by epistaxis, hæmorrhage from the bowels, petechiæ, and extravasations. After death from zymotic diseases, the softening of the organs generally, and particularly of those which are most vascular, is mainly due to the relaxation of the muscular tissue of the blood vessels, is to these organs what the absence of cadaveric rigidity is to the limbs and trunk. The heart is found softened, not from a fatty degeneration, as I have repeatedly assured myself, but from a loss of tenacity of the muscular fibres entering into its composition. It shows the zymotic influence more than other striated muscles, because its unresting activity and rapid nutritive changes call for a full supply of blood, and receive a proportionate supply of its poison.

A materies morbi in the blood, confined there by the membranes of the blood vessels (as all membranes have the power of secerning,) would be in closest contact with the muscular tunic of the smallest vessels, and would exert upon them its paramount influence; but this admission is by no means essential to the theory advanced. The muscular activity of the blood vessels is in constant play and delicate movement of adaptation to the pressure of the blood and the demands of the tissues, so that a poison diffused through the system would make its influence manifest by a disturbance of what is so nicely balanced, and on a tissue of such active nutrition and delicate re-actions.

For these reasons, and abundant circumstantial evidence of their validity, which it would be tedious to detail, I would propose the following definition of fever: *An acute morbid activity of the general circulation and vital combustion, caused by a direct action of the blood-poison upon the muscular tissue of the blood vessels.*

It would require more time than the session could afford to spare, were I to enter at length upon the consideration of the objections which might be presented to this view of the generation of fever; on this head I will therefore be brief.

The School of Medicine at Paris teaches, through Monneret, that "disturbance of the nervous system appears to be the cause of the lesion of calorification and of the other febrile phenomena. How would it be possible to explain, in any other manner, the singular march which intermittents present, commencing and ending at fixed hours, and yielding with facility to sulphate of quinia. In the other pyrexiæ, specific causes appear to play the principal part, by acting on the nervous system. Everything leads to the admission that the vital forces, and the nervous system which is their support, are primarily affected. The study of the causes of the pyrexiæ seems to us to furnish a solid support to this hypothesis. Accordingly we see sometimes a local lesion, as a thorn buried in the flesh, sometimes a septic liquid, the virus of variola, of vaccinia, of marsh miasm, or the spontaneous alteration of the blood, provoke the febrile movement. At another time, it is to the disorganization of an organ that must be attributed the intense fever, which ceases only with the life of the patient." If it is on reasoning such as this that the hypothesis of the nervous origin of fever rests, its foundation is exceedingly insecure. How explain, says Monneret, in any other manner, the phenomena of intermittents? We would ask how the admission affords the least explanation? What recognized law of nervous action explains the commencement and cessation of intermittents at fixed hours, and their prevention by sulphate of quinia? Again, so far from the nervous system being the "support of the vital forces," comparative anatomy, embryology, and teratology teach plainly that the nervous system is an efflorescence, an exalted term, a perfection of organization, by no means fundamental to its manifestations of force. Again, "thorns buried in the flesh" are not a common cause of fever, and the "disorganization of an organ" must inevitably load the blood with the products of its decomposition. The remainder of Monneret's arguments are equally favorable to another hypothesis.

Wood, while lending a guarded adherence to the nervous theory of fever, and the doctrines of Brown and Darwin, confesses their inadequacy to explain some of the most marked characteristics of the febrile state. He says, "Along with the diminished exercise of nervous function, is necessarily a diminution of all the functions dependent on it. We may thus partially explain the condition of the chill, but there is something more which we do not fathom; something, in which the chill of fever differs from other instances of nervous depression. Upon principles which have already been explained, the general prostration is succeeded by re-action, and the fever is thus established. But there is here also something more than re-action. There is the continued action of the cause—a diversified play of sympathies in one case, a widely prevailing influence from some unknown agent in another; and fever is not merely the *resilience* of the bowed-down system." That "widely pervading influence," that "something more than re-action," that "something more which we do not fathom," is a direct influence upon the muscular tissue of the blood vessels. Wood, in combating the doctrines of Cullen, says, "there is no proof whatever of the existence of spasm of the extreme vessels. On the contrary, in the cold stage, they are in a state of collapse from their inability to receive and circulate the blood, and in the hot stage the vessels themselves are dilated, as is obvious from the fullness and redness of the surface." Precisely what condition may be meant here by "collapse from inability to receive and circulate the blood," we will not stop to inquire, but will note merely the admission that impeded transmission of blood by the extreme vessels is a phenomenon of the cold stage, while their increase of caliber marks the pyrexia. Before the bearing of the properties of muscular tissue upon the explanation of the phenomena of fever had presented itself, and while I was interested in merely tracing out the effect of remedies, I had recognized, as a purely muscular property, what has hitherto been attributed to nervous action, the contraction produced by the sudden application of agents capable of relaxing the muscular fibres. In speaking of the effects of immersion of an umbilical artery in water at different temperatures, I noted that an artery raised suddenly to a heat of 120.2°, Fahrenheit, manifested increased rigidity and firmer contraction; raised suddenly to 116.4°, Fahrenheit, it became somewhat contracted, but still more firm on exposure to the cold. Still, I had proved that a gradual elevation of temperature was attended by uninterrupted dilatation till the point of paralysis and complete relaxation was reached at 115°. I had noticed that on admitting suddenly the flow of a heated liquid into an artery the dilatation was preceded by a few minutes of contraction, and the same was true of the action of certain medicinal substances. The following series, taken from among my notes made in the laboratory, presents these facts in a clear light. The amount of a continuous flow from an artery was measured for successive periods of two minutes each. The temperature of the circulating liquid was 110° Fahrenheit.

The following are the amounts:

| | | | | | | |
|---|---|---|---|---|---|---|
| First | period of | two | minutes, | 3 | fluid | ounces. |
| Second | " | " | " | 2⅔ | " | " |
| Third | " | " | " | 2½ | " | " |
| Fourth | " | " | " | 2⅓ | " | " |
| Fifth | " | " | " | 2⅓ | " | " |
| Sixth | " | " | " | 2⅔ | " | " |
| Seventh | " | " | " | 3⅓ | " | " |
| Eighth | " | " | " | 3½ | " | " |
| Ninth | " | " | " | 3½ | " | " |
| Tenth | " | " | " | 4 | " | " |
| Eleventh | " | " | " | 4 | " | " |

Here carbonate of ammonia was added in the proportion of fifteen grains to the pint to the liquid in the upper reservoir.

| | | | | | | |
|---|---|---|---|---|---|---|
| Twelfth | period of | two | minutes, | 5 | fluid | ounces. |
| Thirteenth | " | " | " | 5 | " | " |
| Fourteenth | " | " | " | 3½ | " | " |
| Fifteenth | " | " | " | 3⅓ | " | " |
| Sixteenth | " | " | " | 3⅓ | " | " |
| Seventeenth | " | " | " | 3½ | " | " |
| Eighteenth | " | " | " | 3⅔ | " | " |
| Nineteenth | " | " | " | 4 | " | " |
| Twentieth | " | " | " | 4 | " | " |
| Twenty-first | " | " | " | 4 | " | " |

The temperature of the liquid had fallen five degrees in the forty-two minutes that the experiment lasted, and mono-carbonate of ammonia was evaporating during the eighteen minutes following its appearance in the artery.

That this mode of action is not confined to the blood vessels, was proved in the following manner: From the small intestine of an animal just killed I cut rings by transverse sections. These I adapted to an instrument, by which the ring of intestine was maintained at such a degree of tension by one arm of a lever, to which was attached an elastic band, that the other, or long arm, would mark on the divided arc of a circle the slightest contraction or relaxation of the circular muscles of the intestinal ring. On immersing the ring in water above blood-heat, the long arm of the lever always marked a short period of contraction previous to the continued relaxation, which period of contraction was shorter as the temperature was higher. Similar phenomena of contraction followed the immersion of the ring in solutions, which afterwards steadily dilated it. I found also that by successive increments of heat, as on several speedy withdrawals of the ring from the heated liquid after immersion, I could produce a more continous contraction, from which at last the relaxation was marked and rapid. Should like phenomena occur in the arterioles of a living being, as the study of the circulation of the blood in transparent tissues would lead to believe, a poison thrown suddenly into the circulation, having the property of dilating the vessels, would at first produce their contraction or a stage of chill, while, if its admission were gradual, rigors would not appear. Or, successive increments of poisonous action in the blood vessels, would either produce contraction or hold them in a passive, unchanging condition, till at last sudden and wide dilatation would be accompanied by the explosion of febrile disorder and paroxysmal symptoms in their greatest intensity. We have here an explanation of the so-called accumulation of excitability, the sudden discharge of which results in various paroxysmal attacks, to which the known laws of the nervous system afford no clue; we have at least analogies in the demonstrated laws of muscular action.

That the fever poison is always the same, or that the nervous system does not exert an important influence over febrile manifestations, no one will pretend. But that the cause of fever resides in, or acts through, the nervous system, is, from the preceding considerations, extremely improbable.

I would note also the looseness with which the terms *stimulation, sedation, exaltation and depression of vitality,* are current in the profession. That the necessary consequences of paralysis, or impaired contractility of the blood vessels, should be called stimulation, and the most complete disorder of function be denominated exaltation of vitality, shows an inadequacy or error in the interpretation of symptoms, the consequences of which must be exceedingly unfortunate.

Assimilation, growth and development, are the results of stimulation rather than disassimilation, atrophy and combustion, and an inflammatory affection which leaves an organ shriveled and cirrhosed, or a fever which wastes the system, and leaves it vacillating between life and death, is certainly not an exaltation of vitality.

* "THEORIES," says one of the most cautious of modern physiologists, "give to Science form and motion; serve to bind together facts which must be bound in bundles to be usefully employed; guide and incite explorers in the way of discovery." "Were a theory," says Prof. Grove, "open to no objection, it would cease to be a theory and become a law; and were we not to theorize, or to take generalized views of natural phenomena until these generalizations were sure and unobjectionable—in other words were laws—Science would be lost in a complex mass of unconnected observations, which would probably never disentangle themselves.'' But, from a medical point of view, some justification may be desirable of apparent presumption in offering opinions in opposition to those of widely recognized authority. Fortunately, the question of the nature and causation of fever is, for one of such ancient date and high importance, peculiarly open to discussion. The testimony of George B. Wood, so long our highest authority in general pathology and therapeutics, is, that "there cannot at present be said to be any prevalent doctrine of fever. Each individual has the grounds before him and judges for himself, and probably most persons, seeing a little truth and some error in every exclusive hypothesis, have selected a portion of each and formed a sort of composite opinion, of which even the old Gothic doctrine of the humoral pathologists constitutes a part." Into that composite

---

* The portion which follows of Dr. Stiles' paper was read at the Regular Meeting in June.

opinion it seems to me necessary to introduce another element, one of pre-eminent importance, which has hitherto, as far as I am aware, been overlooked, or which it has been impossible, until recently, to fairly recognize. Moreover, it is not to mere clinical experience that we must look for a revelation of the laws of disease. The laws of chemistry were not discovered in blazing fires or crumbling rocks; the laws of hydrostatics and hydraulics were not revealed in torrents, tides, or ocean-currents; nor those of pneumatics and electricity in winds, whirlwinds, and thunder-storms; much less could it be rationally expected that the laws of pathology should be disclosed amid the much greater complexity and more multitudinous conflict of elements presented to the physician at the bedside of a diseased or dying patient. It is in the laboratory, and by artificially contrived experiments, that the clue has ever been spun and the torch lighted to guide through the labyrinths which hide the arcana of nature. It is to the recognition of this fact, with that other, that the true nature of an object of study is best determined by multiplying the points of view from which it is regarded, together with a general demand by the profession for a pathology which shall be something more than a descriptive catalogue of cases and specimens, that it is owing that those who are older in the laboratory than in clinical experience are heard with willingness in an assemblage of physicians. To oppose the teachings of medical authority may seem ungracious, and may, I am aware, prove disagreeable in proportion to the local sway of the authority opposed; but the trust of faithful study and truthful interpretation of the laws of organization is not therefore to be betrayed.

The theory and definition of fever proposed in a former paper, were the direct and natural result of experimental research, and I deem further experimentation the only proper response to objections which admit of experimental refutation; but enough has already been gained to justify an endeavor to place what has been acquired in such a light as to show its legitimate bearing. By so doing, I shall also better respond to the purpose of arousing discussion, which dictated the announcement of the titles of papers to be presented for a number of successive meetings.

The muscular tissue is one of unstable molecular composition, and of delicate re-actions; it is but reasonable to expect it to be influenced by blood-poisons, or to suffer from impaired nutrition when that fluid is depraved. But I have proved by experimenting on blood vessels devoid of nerves, that their muscular tissue is directly and functionally affected by the composition of the liquids transmitted through them, and that the same liquids exercise a similar influence upon blood vessels which contain nerves. The presence of nerves does not in these instances interfere with the direct action on the muscular tissue. The experimental proof acquired, that substances which have the power of permanently dilating the blood vessels will previously produce their contraction by a direct action on their muscular fibres, renders the intervention of the nervous system in the explanation of the succession of fever to a stage of chill unnecessary. The introduction of the nervous system into the theory is in violation of the first rule of philosophizing, which demands but one sufficient cause. The most characteristic and protracted febrile disorders of zymotic origin are ushered in and accompanied by symptoms which point directly to impaired nutrition of the muscular tissue throughout the body, and *immediately* after death from these disorders the viscera generally, and particularly those which are most vascular, present the characters offered by a cadaver after the rigor mortis of the blood vessels has passed off; they are flabby, and, if abundant hemorrhage has not occurred, are gorged with blood. The heart is found softened, not by a fatty degeneration merely, but by a failure of nutrition of its muscular fibers; its unresting activity has called for an abundant supply of blood; but the blood has become a poison to muscular tissue, and the heart therefore shows the toxic influence more evidently than any other muscular mass. I will not detail the arguments of my former paper on this subject, but will endeavor to offer additional justification of the definition there given of fever. With the groups of symptoms denominated fevers I had nothing to do, but fever was there defined, "*An acute morbid activity of the general circulation and vital combustion, caused by the direct action of a blood poison upon the muscular tissue of the blood vessels.*" Increased activity of the circulation is generally regarded as the fundamental element in the febrile state, the remaining phenomena of fever being its natural results, or mere concomitants. With the words "vital combustion" I associate no theory of animal heat, but simply express the symptom from which fever derives its name. With reference to its causation, I anticipate the following objections:

1st—That the prevalent nervous theory of fever better explains the increased activity of the circulation.

2d—That increased activity of the heart is sufficient to account for the phenomena of fever.

3d—That the definition is too exclusive, in that both the muscular and nervous systems have a share in the production of every fever; or in that, while certain fevers show a direct influence upon the muscular tissue of the blood vessels, others manifest no such influence.

I.—*No nervous theory of fever is adequate to afford an explanation of febrile phenomena.*

Were the nervous theories of fever, those which take precedence of all others in the estimation of the profession, at all adequate to account for febrile phenomena, an opposing theory could hardly claim a notice. The shortcomings of the former would be attributed to the imperfection of our knowledge of nervous action rather than to a defect in the theory itself. There is, however, not only ample room, but a demand for an explanation more in accordance with the increasing definiteness of our knowledge of the distinct powers and reactions of the different tissues and systems of the body. The fact that the activity of the nervous system is due to polar forces, forbids us to base a general disease upon its direct influence in all portions of the system. The poles of a magnet can as readily be supposed to be diffused throughout the whole bar, as the nervous system to produce direct excitement of the circulation in every blood-vessel of the body at the same time. The nervous system being an apparatus of relation, it is not within its province to cause directly a general disease of circulation and nutrition. It is on this character of nervous action that depends the therapeutic method of revulsion. Revulsive measures owe their efficacy to the facts involved in the following statement of Wood, that "there is in the human system only a certain capacity of nervous action, and a certain amount of blood. When either the former or the latter is strongly directed to a particular part of the body, there is a tendency to its diminution elsewhere." No one will pretend that a different system of nerves is involved in the disease and in the counter-irritation by which it is relieved; and no physician, who has been able to draw any substantial and well-grounded conviction from his medical experience, will allow his faith in the value of counter-irritation to be shaken: he must throw aside the nervous theory of fever should it be found to conflict with so solid a conviction. But those cases to which counter-irritation is least applicable are those in which the grade of fever is highest. Such cases are even aggravated by revulsive measures. If we have not learned to "bring the system to the blistering point," by venesection and antiphlogestic remedies, when high fever accompanies an inflammation, it is not the fault of our teachers. Were the increased activity of the circulation in such cases due to nervous influence, the fever would be relieved by a concentration of nervous energy in the part counter-irritated, or at least diminished in proportion to that derivative influence, for there is in the system "only a certain capacity of nervous action." The counter-irritation may be severe, but the fever does not in the least abate. Idiopathic fevers likewise will run a steady course, in spite of endeavors to concentrate the blood and nervous energy in any particular part of the body. They will likewise run their course in spite of powerful remedies directed to the nervous system, and exerting upon that system a full measure of their characteristic physiological influence. Could fever thus evade all the influences acting upon the nervous system if it had its immediate origin in that system? But there are other and equally weighty arguments against such an admission. Bernard discovered that a persistent local hyperæmia and elevation of temperature without inflammation—in other words, circumscribed febrile phenomena—would be produced in various parts of the system by section of the sympathetic nerve supplying the blood-vessels of those parts, and he traced these phenomena to a dilatation of the blood-vessels dependent on their muscular tunic. Whatever support this discovery may seem to lend to a nervous theory of fever, it must be remembered that it proves that elongation of the circular musular fibers of the blood-vessels is one link in the chain of causation, and if it can be shown that a direct influence through the blood upon the muscular tissue better explains the phenomena of fever, the introduction of the nervous system into the problem is an unnecessary complication. But, in the experiments alluded to, increased heat and vascularity follow closely the section of the nerve; whereas, in cases of severe nervous injury, hours elapse before the fever is developed, which, when developed, bears no relation to the severity of the symptoms of collapse, and may be violent when there has been no marked shock or nervous depression. No pathologist has ever attempted to predict the gravity of a traumatic fever from the amount of injury recognized to have been sustained by the nervous system. Again, death from protracted febrile disorders, as typhoid and typhus fevers, leaves the nervous system in a state of integrity which contrasts strongly with the softened and disorganized condition of the vascular viscera of the thorax and abdomen; the brain and spinal cord are neither softened nor discolored, nor in an appreciable manner diseased in structure, as the direct result of the most protracted fatal febrile disease. I have carefully examined quite a number of such cases without being able to detect any appreciable lesion. There is, therefore, no proof that the nervous system is affected in the production of febrile disorder, while the proofs of impaired muscular nutrition are very decided.

II.—*Febrile phenomena cannot be explained by increased activity of the heart.*

Boerhaave and Broussais taught that increased activity of the heart was the immediate cause of fever. One of Broussais' "Four Hundred and Sixty-eight Propositions" is, that "fever is always the result of an irritation of the heart, either primary or sympathetic." That excitation of the heart is one link in the chain of causation is very evident; but that over-excitement of the heart is of itself sufficient to generate a fever, few will be found willing at the present time to admit. Without dilatations of the arteries and arterioles (for the true capillaries of a single elastic tunic are not contractile, and these only are capable of yielding to the force of the heart), the heart has little power to increase the activity of the circulation. In such vessels excess of pressure may produce varicosities, or give the vessel a beaded appearance, but the faculty of transmission of blood will not thereby be augmented. Pressure is a much less important element than calibre in determining the rate of flow in inelastic tubes like the finest arterioles. In experimenting on this subject, I obtained in two cases the following results: An inelastic tube, which discharged nine fluid drachms in thirty seconds, under a pressure of one foot of liquid, discharged thirteen and a half fluid drachms under a pressure of two feet; seventeen drachms under one of three feet; and twenty under one of four feet. A tube which discharged seventy minims in thirty seconds, under a pressure of one foot of liquid, discharged a hundred and ten minims under one of two feet; a hundred and forty-five under one of three feet; and one hundred and seventy under one of our feet. It required in these experiments about three times the pressure to double the rate of flow. But the pressure in the finest arterioles and capillaries varies within a very narrow possibl range. Bernard gives the following figures in proof of the narrow range of degrees of constant pressure within the arteries of animals of very different size. The fixed constant pressure in the carotid of a horse, a dog, and a rabbit, were respectively 110, 103, and 95 millimetres of mercury. The osciltations, however, due to the pulsations of the heart were much wider, being 65, 12, and 5 millimetres respectively; but even in the horse, whose disproportion in size to that of man is so great, the greatest momentary tension under the pulsation of the heart is little more than one-half of the constant pressure. In a dog, the constant pressure in whose carotid was 110 millimetres of mercury, and the maximum 165, the opposite carotid was tied, when the constant pressure rose to 165, and the maximum to 200. The maximum momentary pressure in this extreme exaltation of tension was not double the normal minimum pressure. But the force of the heart is lost in the arteries as they approach the capillaries, and the possible variations of pressure are there still further reduced; the heart's energy is too far spent in overcoming the resistance to the flow of blood in the successive ramifications of the arterial tree, with their barriers of friction and anastomosis, to influence the inelastic tension of the circular fibers of the blood-vessels. The height to which a column of mercury is raised in a vein is at best but a small fraction of the pressure in the corresponding artery, when the valvular arrangement of the vein is not brought into play by the activity of the voluntary muscles.

If, however, the diameter of a capillary tube be doubled, the quantity of liquid discharged under a given pressure will be sixteen times as great. If a capillary tube by dilatation become one-fourth larger, it will transmit considerably more than double the amount of blood (512 to 625.) It is thus evident, on the simplest physical principles, how small a proportion of the increased activity of the circulation, from whatever cause arising, can possibly be due to increased activity of the heart, compared with the influence exerted by the increased calibre of the blood vessels, as regulated by their muscular tunic.

III.—*The nervous disorders presented by every case of fever are secondary or coincident, not fundamental.*

That a poison in the blood, or an alteration of its composition which has the power to affect the nutrition and activity of the muscular tissue, would exert a direct influence on some portion of the nervous system also, is a very rational presumption; that the nervous system should feel and manifest the affection of the muscular system is inevitable; both these admissions are entirely consistent with the theory advanced, which, nevertheless, requires a direct influence on the muscular tissue of the blood vessels as the fundamental element in every case of fever. The arguments from counter-irritation, and from the relational office of the nervous system, apply to all cases of fever, whatever may be their distinctive peculiarities, and point to a common mode of generation independent of nervous origin. The nervous system is not constituted in dependence upon positive amounts of generally distributed force, but re-acts to variations in amount and distribution. Its phenomena do not essentially vary in the robust and plethoric, and in those whom chronic disease has rendered almost exsanguined. A temperature of forty degrees to a hand at sixty is no more sensible than one of sixty to a hand at eighty. Hence the various forms of tolerance to which the nervous system may be subjected. Abnormal distribution to it of blood may produce the most violent symptoms, as in insanity, epilepsy, and eclampsy. So delicate is the re-action of the nervous system of the highest organisms to foreign substances reaching it through the blood, that the resulting nervous phenomena present the greatest variety—even idiosyncrasies, those disheartening nervous problems, are thus rendered possible.

The material cause of the diseases known as fevers is of very various origin, and doubtless of very diverse molecular constitution. How different the source of the poison in the simple irritative fevers, in intermittents and remittents, in yellow fever, in typhus and typhoid, and in the exanthemata! Yet the description of the symptoms which usher in one of these is applicable alike to all, and in our most authoritative treatises on special pathology the expressions, "general discomfort, weariness, and languor, deficiency of appetite, furred tongue, disordered taste, soreness or numbness of the limbs, pain in the back, headache, mental depression, irritability, want of sleep," or their equivalents, form the common tableau of prodromata. How different this from the distinct and specific symptoms by which we recognize the very commencement of medicinal or morbid action on the nervous system! Lastly, the fever formed defies all attempts at arrest, or essential modification, by remedies addressed to the nervous system. The cause of the fever may lie, indirectly, in severe mental strain, or in some overpowering sorrow; the cause may persist in all its force when the fever is aroused, and often happily removes the system from nervous sway, and saves a life which would otherwise fail to sustain the nervous tension. A blood-poison has been produced through the agency of the nervous system, which, by acting on the muscular tissue, has released the system from nervous control, and generated the fever. The admission of such an element is a necessity, for "fever is more than the resilience of a bowed-down system," and is warranted by the fact that the production of vitiated fluids through nervous agency is a matter of not unusual observation.

I have reserved till now the nearest approach to experimental demonstration which I have been able to obtain, that in the fevers the muscular tissue is directly affected, in order to give the facts their due prominence. This was accomplished in the following manner:—Portions of umbilical arteries from a cord just divided were dissected out, as is easily done, and attached to ligatures. Blood was then drawn by cupping from a patient suffering from typhus fever, either simple, or complicated with inflammation. The blood was defibrinated and placed in one or more test-tubes. Healthy blood was treated in the same manner, and the prepared portions of umbilical artery were suspended in the diseased and in the healthy blood, and exposed for a given time to a temperature of 100° Fahrenheit. In each experiment, after an hour's immersion, it was easily recognizable which portions of artery had been suspended in the febrile and which in the healthy blood, by the flaccidity, discoloration, and lack of vitality manifested by the former, and the healthy appearance and re-actions of the latter. Such results as this are of more value than long and labored arguments, and will, I hope, serve as an excuse for shortcomings in that respect.

